# Structured care after a DSD diagnosis in childhood: a mixed methods evaluation of the Empower-DSD program

**DOI:** 10.3389/fped.2025.1488411

**Published:** 2025-03-24

**Authors:** Katja Wechsung, Louise Marshall, Martina Jürgensen, Sabine Wiegmann, Ute Kalender, Manuela Brösamle, Gloria Herrmann, Olaf Hiort, Gerda Janssen-Schmidchen, Annette Richter-Unruh, Martin Wabitsch, Charlotte Wunn, Thomas Keil, Uta Neumann, Barbara Stöckigt

**Affiliations:** ^1^ Department for Paediatric Endocrinology and Diabetology, Center for Chronically Sick Children, Charité—Universitätsmedizin Berlin, Corporate Member of Freie Universität Berlin, Humboldt-Universität zu Berlin, Berlin Institute of Health, Berlin, Germany; ^2^ Division of Paediatric Endocrinology and Diabetes, Department of Paediatrics and Adolescent Medicine, University of Lübeck, Lübeck, Germany; ^3^ Institute of Social Medicine, Epidemiology and Health Economics, Charité—Universitätsmedizin Berlin, Corporate Member of Freie Universität Berlin, Humboldt-Universität zu Berlin, Berlin Institute of Health, Berlin, Germany; ^4^ AGS-Eltern- und Patienteninitiative e.V., Schönkirchen, Germany; ^5^ Division of Paediatric Endocrinology and Diabetes, Department of Paediatrics and Adolescent Medicine, Hormone Center for Children and Adolescents, Ulm University Medical Center, Ulm, Germany; ^6^ SHG Interfamilien, Dresden, Germany; ^7^ St. Josefs Hospital, Pediatric Endocrinology & Diabetology, Ruhr-University Bochum, Bochum, Germany; ^8^ Intergeschlechtliche Menschen e.V., Hamburg, Germany; ^9^ Institute of Clinical Epidemiology and Biometry, Würzburg University, Würzburg, Germany; ^10^ State Institute of Health I, Bavarian Health and Food Safety Authority, Erlangen, Germany

**Keywords:** DSD, sex development, shared decision-making, structured care, information transfer, support, guideline

## Abstract

**Introduction:**

Differences of sex development (DSD) encompass several rare diagnoses with medical and social implications. If a child is suspected of having DSD, timely and comprehensive information to the family is crucial for an undisturbed parent-child relationship and a good outcome. Providing information and competent care for a child with DSD is challenging for medical staff and parents, especially at the beginning of care, when many diagnostic results are still pending. The Empower-DSD information management program provides a structured multidisciplinary care and information exchange for children and their parents in the first 8–12 weeks after presenting to a specialized DSD center.

**Methods:**

From June 2020 to August 2022, 51 families completed the structured care pathway in 4 DSD centers in Germany as part of the government-funded Empower-DSD study. The program was evaluated with a quantitative and a qualitative approach. Diagnosis, age of child, total duration, number of appointments, and completed elements of care were documented. Semi-structured guided interviews with parents, peers and professionals were used to explore expectations and the experience of the involved stakeholders.

**Results:**

Care elements were documented in 11 children with congenital adrenal hyperplasia (CAH) and 28 children with other DSD-diagnoses (chromosomal DSD; 46, XY-DSD; 46, XX-DSD) with a mean age of 1.8 years (0–18 years). In total 45 people were interviewed. The information management program alleviated stress and uncertainties for parents and encouraged a trusting relationship with the DSD team. Professionals rated the developed materials as a valuable tool to provide consistent and thorough care. Parents underlined the importance of the early access to specialized DSD teams, a clear and open communication and the reassuring attitude of professionals in DSD care. Parents and professionals stated that the program required time and resources and would prefer an individualized approach instead of a predefined duration.

**Conclusion:**

The structured, multidisciplinary support within the first weeks after a DSD diagnosis was perceived to be of high quality by all stakeholders involved. Information on the nature of the decision-making process and peer narratives could be added to the information material.

## 1 Introduction

Differences of sex development (DSD) encompass congenital conditions with an associated uncommon development of chromosomal, gonadal or anatomic sex. Individual preferences exist on the appropriate designation (1). Similar alternative terms such as variation of sex development or a condition specific term are used (2). According to the 2006 Chicago Consensus Guideline, diagnoses can be grouped into chromosomal DSD (such as Turner Syndrome and Klinefelter Syndrome), 46, XY DSD or 46, XX DSD (3, 4). Androgen excess due to congenital adrenal hyperplasia (CAH) leads to a form of 46, XX DSD. In DSD-diagnoses, children can present with ambiguous genital appearance, endocrine and/or reproductive challenges or possible syndromic features.

Many parents/caregivers (will be mentioned as parents throughout) are anxious and concerned after their child is diagnosed with DSD (5, 6). They anticipate there will be stigma and barriers for the emotional wellbeing of their child and search for optimal care and treatment (7).

In the first weeks after the diagnosis, parents of infants face complex decisions about their child's health and future (8). This might include the decision for a name and a possibly provisional gender for the rearing of a newborn child, education of family/friends or the wish for medical interventions. Given these concerns, a quick referral to specialized care and availability of high-quality information are important for families (9). Additionally, there is limited evidence on the long-term outcomes of different choices that can support decision-making (10). Decisions heavily depend on the personal values and preferences. Young children are dependent on their parents’ decisions, which are based on the parents’ values (11, 12). Parents might ask for early surgery to modify genital appearance as one option of treatment. In Germany, since 2021 the law prohibits deferrable surgery on sex characteristics in children with DSD until they can provide informed consent (13). The capacity to give consent is defined in an assessment by the specialist staff. The age of the child is explicitly not specified. However, the law only applies to children under the age of 18. If the parents request a treatment that alters the appearance of sex characteristics before a child can give informed consent, it can be performed only after an evaluation and statement of an interdisciplinary committee experienced in DSD and a family court approval (14).

Until adolescence, parents play a key role in decision-making. For example, children of primary school age may struggle to fully understand the meaning of living with a DSD (15). Young people can be shaken in their identity by a new diagnosis and may find it difficult to actively participate in making decisions and communicating with others (e.g., peers) about the condition (15).

As such, providing the family with sufficient support and information to navigate the decision-making process and promoting the education of the child is essential to achieve a positive outcome (16, 17).

Historically, the gender of rearing and any associated gonadal or urogenital surgeries were determined by the medical team in a paternalistic way and the diagnosis was concealed from the child (18). Reports of harm due to early genital surgery and reduced wellbeing of adult individuals with DSD led to a paradigm shift in DSD care (19). Family centered supportive interdisciplinary care, condition openness, and informed shared decision-making are now considered important elements for a successful clinical management of children with DSD (20, 21). The authors of the 2006 guideline and the 2016 updated consensus statement emphasize the importance of care in specialized DSD centers (3, 4). A multidisciplinary teams (MDT) consisting of professionals from pediatric endocrinology, pediatric radiology, surgical subspecialties (pediatric surgery and/or pediatric urology and/or pediatric urology), genetics, psychology and social work offers medical expertise and psychosocial support. Peer support (contact to families with children with DSD or adult members of self-help groups) can provide experience, emotional and practical help to children with a recent DSD diagnosis and their parents (22).

The education of the family is a primary goal of the multidisciplinary care at the DSD Centers and a prerequisite for shared decision-making (23). Imparting knowledge on the complex medical and social aspects of DSD while waiting for the results of the diagnostic process is a challenge for children, their parents, and the MDT in the first weeks of care (24). The central aim of the German, government funded Empower-DSD study (01VSF18022) is a consistent, structured education of children with DSD and their parents, to promote informed decision-making in the interests of the child and the empowerment of the child and the parents. This program aims to encourage the best possible and self-determined development of children and to increase their resilience. Within the Empower-DSD study a structured care and information management process was developed (Empower-DSD information management program) (25, 26). It standardizes the multidisciplinary care of children according to present guidelines and specifies the information exchange in the first weeks after first presentation to a DSD center.

A detailed description of the developed materials and care elements of the information management program has been published (27). The developed materials were implemented and evaluated in four DSD centers in Germany. This paper on the project evaluation gives insight into the expectations and the experience of different stakeholders in the first weeks after a DSD diagnosis.

## 2 Materials and methods

### 2.1 Study design

Empower-DSD is a prospective longitudinal, mixed method, non-controlled multicenter study with the intervention Empower-DSD information management program.

#### 2.1.1 Intervention (Empower-DSD information management program) and participants

Children of different age groups (newborn to adolescent child) and their parents were eligible to participate in the standardized Empower-DSD information management program. We sought to enroll 30 children and adolescents with a genital variation and their parents in the information management program. Families were invited to participate if, at their first appointment at the DSD clinic, the child was suspected to have a DSD. Children with Turner and Klinefelter syndrome were only eligible if they presented with urogenital atypicality. Parents and children from the age of 6 years had to give written consent/assent.

The program was implemented in 4 participating DSD centers in Germany (University hospitals in Berlin, Bochum, Lübeck and Ulm), based on the developed materials described previously (27). A guideline for the DSD teams outlined structural requirements, e.g., professions to be involved in the MDT and defined the duration of the information management program as 8–12 weeks after the first appointment of the family in the DSD center.

A checklist was used to track the implementation of care elements: (1) introduction of team members; (2) diagnostic process; (3) discussion of results and medical information; (4) psychosocial care of parents and children, (5) peer support, (6) team meetings, (7) case conferences and (8) feedback to the primary care provider. The families were handed the folder “my record” at the first visit, containing information on age-related medical and psychosocial issues. The family was encouraged to use the folder as a place to collect medical results and document personal thoughts regarding the DSD diagnosis or decision-making.

In addition to a family resource, a booklet with information for medical staff not specialized in DSD was developed during the Empower-DSD study to improve the care in the first days (27). A digital copy of the booklet was distributed throughout the German societies for gynecology and neonatology in 2020. All developed materials are available in German for free download on the Empower-DSD website (28).

#### 2.1.2 Research team

The research team consisted of peers and professionals. Representatives of self-help groups for CAH (AGS-Eltern- und Patienteninitiative e.V.) and 46, XX-/46, XY-DSD or chromosomal DSD (SHG Interfamilien; Intergeschlechtliche Menschen e.V.) were referred to as peers. Professionals consisted of members of MDT (psychologists and medical doctors) in 4 DSD centers in Germany, and staff of the institute of social medicine, Charité Berlin (a medical doctor and a sociologist).

### 2.2 Evaluation

The mixed methods approach of this study followed a parallel design (quantitative and qualitative data were collected in parallel). The research team integrated results in a data- and results-orientated manner in a communicative and collaborative process using triangulation (29). The different perspectives allowed a comprehensive understanding and interpretation of the results.

The implementation of the developed structure and materials of the information management program were analyzed in a quantitative descriptive approach. The MDT documented the information management process in a checklist (27). Diagnosis (categorized into 46, XX-/46, XY-DSD, chromosomal DSD or congenital adrenal hyperplasia, CAH), age of child, and documentation of the information management process in the checklist (total duration of the information management program, number of appointments, completed elements of care) were analyzed with descriptive statistics.

Qualitative data was collected via semi-structured interviews exploring hopes, expectations, and the experience of the stakeholders involved in the program. Families, peers, and professionals who worked on the implementation or development of the information management program were invited to participate in interviews. Staff of the institute of social medicine, Charité Berlin prepared drafts for the interview guides. All members of the research team (peers an professionals) discussed and revised the drafts in a participatory manner. Details on the interactions and negotiations that took place during the development process have been published elsewhere (30). The interviews were planned to be held face-to-face or by telephone during or after participation in the information management program. The interviews were digitally recorded, transcribed pseudonymously, and coded, categorized and analyzed on the basis of a qualitative content analysis (31). The coding was carried out inductively (from the data material) and deductively (according to the research question and the interview guide). Data analysis was carried out using MAXQDA® software. Two scientists (a medical doctor and a sociologist) with expertise in qualitative research carried out the data collection and analysis. The results were regularly discussed within the Empower-DSD research team and the interdisciplinary, qualitative working group of the qualitative Research Network, Charité—Universitätsmedizin to enhance intersubjectivity.

### 2.3 Ethics

The study was conducted in accordance with the Declaration of Helsinki and was approved by the ethics committee of Charité Universitätsmedizin Berlin (EA2/238/19; 4 March 2020) and the local ethics committees of the participating study centers.

## 3 Results

From June 2020 to August 2022, during the COVID-19 pandemic, 51 families completed the information management program. The pandemic did not restrict patient recruitment or the implementation of the information management program in the participating study centers. Some families were offered appointments for psychosocial counseling via telemedicine. Team meetings in the DSD center as well as case conferences between the four DSD centers were held online. Contrary to the inclusion criteria, a child with Turner Syndrome (Karyotype 45 X) was included without genital ambiguity but only according the DSD-classification in one participating DSD center and the parents were interviewed. We did not exclude the participant from the analysis.

### 3.1 Results of the quantitative evaluation

Information on 40 families were available for quantitative evaluation from the checklists completed by the DSD teams. Two families dropped out of the study and for 9 families the checklists were not returned. Of the children analyzed, 28 (70%) had the diagnosis 46, XX-/46, XY-DSD or chromosomal DSD and 11 children the diagnosis CAH. For one child, the suspected diagnosis was not reported. In 24 children, a genetic cause for the genital variation was identified during the information management program. For six children no information on genetic results was available.

The mean age of included children was 1.8 years (0–18 years). The median age was 0 years (IQR 0–1). All children with CAH were diagnosed when they were newborns. In the group of children with 46, XX-/46, XY-DSD or chromosomal DSD the majority presented within the first 2 years after birth (22 children, 78%).

On average, the duration of the information management program was 6 months (range 1- 20 months). Appointments within the defined timeframe of 8–12 weeks were documented for 19 families (48%), while 12 families (30%) needed 12 months and 8 families (20%) longer to complete the information management program. The mean number of appointments in the DSD centers were 4 (range 1–15 appointments) and varied between the centers (Berlin 6, Bochum 5, Ulm 5 and Lübeck 2 appointments on average).

No difference was observed in the mean number of appointments regarding the diagnosis [mean 4 (range 1–9) appointments for 46, XX-/46, XY-DSD or chromosomal DSD, and mean 5 (range 1–15) appointments for CAH].

In Table 1 the completed care elements documented in the checklists are displayed. The DSD teams documented the first visit, psychosocial and medical counseling, diagnostic process and the case conference within the DSD center for the majority of the families. Peer counselling for the child or family, discussion of social and legal issues, and feedback to the primary care provider were documented less frequently.

**Table 1:** Care elements documented as completed by the MDT in the DSD center in percentage of all included families.

Care elements of the Empower-DSD information management program	Documented in the checklist (*n* = 40)
First visit (explain goals of the information management program and concept of peer counseling and psychosocial care, introduce team members, hand out “my record”, outline diagnostic process)	80%
Medical information	100%
Psychosocial care	93%
Social and legal issues	34%
Peer counseling	28%
Diagnostic process	100%
Case conferences	
Team in the DSD center	100%
Cross-center between 4 participating DSD centers	60%
Feedback to the primary care provider	10%

### 3.2 Results of the qualitative evaluation

The interview guidelines were developed in a participatory manner (30) and resulted in the following topics:

(1) Experience of care in the Empower-DSD information management program
(2) Subjective perceived effects of the information management program
(3) Expectations and wishes for the information management program
(4) Dealing with the diagnosis/suspected diagnosis (parents only)
(5) Working with people with DSD (professionals, peers only)

#### 3.2.1 Sample

In total, 45 people (15 parents, 12 peers and 18 professionals) were interviewed (Table 2). Most of the interviews were completed individually; however, five parents were interviewed in pairs. The majority of the children in the families interviewed were under the age of one, only two children were older (7 and 14 years). No children were interviewed. All interviews were conducted by telephone due to the Covid-19 pandemic.

**Table 2:** Characteristics of interview partners.

Diagnosis of child	Interviewed persons				
	Parents	Peers	Professionals		
			Medical doctors	Psychologists	Other specialist staff^a^
CAH	6	3	8	5	5
Turner syndrome	1	3			
Klinefelter syndrome	–	1			
46, XX-/46, XY-DSD	8	5			
Sum of interviews	15	12	18		

^a^ Members of the MDT with the following professions: Pedagogue, scientist, study nurse, medical clerk, and medical assistant.

#### 3.2.3 Central categories

The qualitative analysis revealed the following central categories:

1. Expectations (deductive)
2. Experience and perceived effects of care in the information management program (deductive)
3. Early connection to specialized care/diagnosis outside a DSD center (inductive)

##### 3.2.3.1 Expectations

The main focus of the families was to receive the best possible care and support for their child. To cope with the unexpected diagnosis, they needed a “roadmap” to clarify emotions. Families wished to address questions and concerns in calm conversations with healthcare professionals. They wanted decision-making processes that were focused on participation and empowerment.

The professionals expected improved interdisciplinary cooperation and knowledge exchange through the structured process of the information management program, e.g., in case conferences. They hoped that the collaboration between the centers would be integrated into routine care after the study. In general, there was hope that families would receive better care, which would promote participation and improve decision-making as well as empower families.

Peer counselors consistently expressed the wish that self-help groups should be more involved in the counseling of families.

“Above all, [I expected] that the parents would benefit from it [the information management program], making part of the decision-making process easier for them and thereby more understandable for the whole family.” (Professional, medical doctor)

##### 3.2.3.2 Experiences and effects of care in the information management program

Families emphasized the importance of feeling supported and having enough space and time to address their questions, concerns and needs, regardless of the child's age. They appreciated an empathetic, calm approach. Psychological support promoted the acceptance of the diagnosis. After an initial feeling of being overwhelmed, the families found relief in the structured care at the DSD centers and benefited from competent guidance, understanding, and a clear plan. A supportive attitude from the staff as well as open and clear communication contributed to a trusting relationship. The families appreciated tailored multidisciplinary care, including early contact to peers. Nevertheless, they perceived the frequent appointments, especially in the initial phase, long journeys to the specialist center, and the considerable amount of information as stressful and unsettling. New information, even when explained in detail, raised new questions and concerns regarding the child's future. Conflicting opinions or recommendations from different members of the team and uncertainties in communication led to doubts among parents.

"Yes, at the very beginning, all the questions we had about what we had to look out for now (..) they took away all our initial worries (..) also we didn't really know where we belonged.” (Mother of daughter with CAH, 4 months)

“I had to sort myself out a bit, because at the beginning we were bombarded with a lot of information and various study enquiries. (..) [Our doctor always] (..) took time for this conversation and also asked (..) if we had any questions and (..) that (..) brings a sense of relaxation into the whole thing.” (Mother of daughter with 46, XX-/46, XY-DSD, 13 years)

Professionals emphasized the time it took to respond comprehensively to the families’ questions and needs. They stressed the satisfaction that resulted from the intensive support they could offer the families. The information management program provided a clear framework for the implementation of existing DSD guidelines. Professionals rated the folder (“my record”) positively for its structure and transparency. Case conferences were seen as helpful, but also as time-consuming. Initially, the professionals felt stressed by the demands of the information management program but gained surety with more routine. The professionals perceived a period of 8–12 weeks as to pressurized and desired a more individualized timeline.

"I think it’s an advantage that there is a kind of checklist for the doctors so that they really think of everything and don't forget to address important points. (..) I also think this folder that we have is very good. Simply that it also gives you the opportunity now, when the child is still small (..) to be able to follow the process later. ‘What did my parents do? Why did I go to the doctor so often?’ That (..) is a nice way to summarize everything, helping the child to better understand their history later on.” (Professional, medical doctor)

The peers appreciated the comprehensive exchange of information and the multidisciplinary support network for families in the information management program. They welcomed the normalizing way of dealing with the diagnosis. The structure of the information management program facilitated standardized care and the work of the peers. The folder “my record” was valued as providing structure and strengthening for the family. Peers considered their work in self-help groups and peer counselling to be important. In particular, at the time of diagnosis, they could support families with their experience and knowledge. The contact between families and peers was often established by the families themselves and not by the MDTs. However, many families contacted peers at a later stage.

"I can simply give important tips for later and (…) they have corresponding partners (…) to exchange, how to learn to deal and to live with it [the diagnosis]. (…) then the first fears can be mitigated or also intercepted.” (Peer counselor, CAH)

##### 3.2.3.3 Early contact with the center/diagnosis outside the center

Parents emphasized the importance of early contact with a specialized center. A diagnosis in a tertiary center was perceived as a traumatizing experience, especially during the most vulnerable time around the child's birth. They reported unsympathetic encounters, a lack of specialist knowledge, and the feeling of isolation and strain. The empathy of psychologists outside the centers was positively recognized. Furthermore, geneticists were described as professional and socially competent. Families considered themselves lucky if they received specialized care from birth on and were willing to travel long distances to receive it. Contact with peers was also seen as very helpful. Professionals emphasized the importance of the MDT in the specialized center, while peers appreciated that the families were given a sense of normality.

“From my own experience, I can say that it’s good to contact other families or intersex people early on, because they often simply say ‘everything is okay, it’s nothing bad, it’s not a disease, your child is fine’. [That] brings some calm. I would also recommend (..) that you go to a specialist team where there are people who do nothing else all day, i.e., who deal with intersex people or children. So that this sensation is a little less sensational and the focus is on the person and not on the diagnosis of intersex.” (Mother of daughter with 46, XX-/46, XY-DSD, 8 months)

### 3.3 Triangulation

The quantitative and qualitative results complemented one another. Both the checklists and the interviews showed that the treatment in the DSD clinics was comprehensive and multidisciplinary. In the interviews, it was apparent that the information management program was rated positively by families, professionals, and peers, but was also perceived as time consuming and demanding. Although the timeline for program completion was initially 8–12 weeks, results revealed that a more individualized timeline was appreciated.

The case conferences were regarded as valuable for interprofessional exchange, yet also as time consuming and occasionally difficult to organize. Peer counselling was documented in one-third of the checklists of the included families, aligning with peer interview statements that parents often approached them after some time had passed since the initial diagnosis and with the parents interview statement that contact to peers was not only arranged through the MDT. The discussion of social and legal issues was considered important in the interviews, but was only documented in the checklists for one-third of the families.

## 4 Discussion

The Empower-DSD information management program offered a structured multidisciplinary care and information exchange in the first weeks after presentation to the DSD center. Four German DSD centers successfully implemented the program. Thereby, standardizing care and providing a MDT that includes psychosocial support in the first weeks after diagnosis, which has been a challenge in the past (32, 33). To the best of the authors’ knowledge, the information management program is the first evaluated clinical care pathway for children with DSD that translates the existing clinical practice recommendations into a clinical process (34).

The results of the qualitative and quantitative evaluation gave insight into the expectations of different stakeholders in the first weeks after a suspected DSD diagnosis. Parents wished for support and orientation. They wanted their feelings and concerns to be considered without time pressure. Peers wished for the integration of self-help groups. Professionals expected improved interdisciplinary networking and care for families (enhancing shared decision-making, empowerment). These results are consistent with previous surveys (27, 35).

In the study the effects were analyzed of standardized care and the experience of structured information exchange on the participants. The information management program provided information and encouraged a trusting relationship with the MDT. Thereby, it alleviated stress, worries, and uncertainties for families in the first weeks after a new DSD diagnosis. Parents, peers, and professionals gave positive feedback on the developed materials.

### 4.1 Time and resources

In the qualitative and quantitative analyses it was shown that the time and resources required by the information management program were demanding for families and professionals. The checklist was used for the majority of the included families. It structured the care process and improved interdisciplinary cooperation. However, completing the checklist was time-consuming. This could explain why 9 checklists were not available for evaluation.

The duration of the initial information transfer varied between the families and location of the DSD centers. The Empower-DSD study defined a duration of 12 weeks for the information management program. Half of the included families exceeded this time. This is consistent with the experience that families may not be ready to take on information without time to get used to the diagnosis and its psychosocial implications (36). Moreover, it takes time to schedule diagnostic procedures, multidisciplinary counseling, and to wait for results (e.g., genetic testing). Families experienced the frequent appointments at the specialized center as stressful and travelling to the DSD center as challenging. In the MDT, the individualized care for families required multiple resources such as time for interdisciplinary exchange, for appointments with the family, flexibility, and accessibility for questions in-between appointments. DSD centers should set up structures to be equipped for these challenges. An open time frame without deadline for initial information transfer and decisions may help to reduce the stress.

### 4.2 Ongoing information transfer

The information management program can initialize an open communication process about diagnosis and care. The majority of the children included in this study were infants. A challenge is the ongoing transfer of information to the growing child (5). The age at which children can be involved in informed decision-making differs and it is important to bring up information repeatedly (4). The continuous documentation of discussed topics and findings of various diagnostics in the folder “my record” can give the families the feeling of being informed. It can be helpful later for the time when the child can make its own decisions or for the transition of adolescents to adult care. For the young adult, the values of the family and the influences of social and legal conditions remain traceable through the folder. DSD-specific patient training programs at different ages can complement the information transfer during consultations at the specialized DSD centers (25). Training programs were developed and evaluated within the Empower-DSD study and curricula are available in German upon request (26). Follow-up surveys should assess the long-term ongoing use of the folder “my record” by the participating families and its role in the information transfer. In view of rapidly changing medical information, social values, and legal issues, “my record” needs to be updated regularly to remain a valuable tool in the future. Ideally, the health systems should install and finance permanent structures to ensure a high quality of the information tools.

### 4.3 Early access to specialized care

The time before the start of specialized care proved to be particularly stressful for parents. The positive feedback from parents on certain professions, such as geneticists and psychologists, may reflect the importance of a reassuring and calm attitude during the contact with patients with a rare diagnosis such as DSD. Geneticists are used to counseling families with rare genetic variations and to convey information in a non-alarming way. Training programs for professionals in maternity clinics or gynecological practices could improve care after a new DSD diagnosis. Training might be focused on attitude and communication rather than on diagnostic procedures. In order to ensure the earliest possible connection to a DSD center, national structures may help. One such example is the project DSDCare, which has launched a website to compile relevant information on DSD, peer group contacts, care pathways, and patient-oriented processes in the participating centers in Germany (23, 37).

Although a booklet with information for non-specialist DSD staff was developed and distributed as part of the information management program, this intervention was not further evaluated (27). Future studies should focus on the period prior to specialized care in the DSD center. An evaluation of the effect of an information booklet for non-specialist DSD staff could provide further evidence and opportunities to improve early care in tertiary centers. Furthermore, the materials developed could also be useful for prenatal education and counseling of parents whose child is suspected of having a DSD.

### 4.4 Common language in the team and challenges in decision-making

Contradictory statements from health professionals were found to confuse parents. A close exchange within the MDT can help to prevent additional uncertainties for the parents. Care should be taken not to persuade the family for one choice (16). However, the wish for a common language of all professions involved might also reflect the complexities of DSD decision-making. According to the literature the majority of parents of children with DSD report decisional conflict (8). Social and religious background largely influence decision-making (38). Decisions are made in a deliberative process and often require compromising one desired outcome in favor of another (21). Changing atypical genital appearance to prevent future stigma or preserving the physical integrity of the child are opposing decisions. Parents and the MDT have to weigh the benefits and harms of each decision and explore the role of personal preferences in decision making.

Information for the families to understand the challenges of the decision-making process and material for decision coaching could be added to the folder “my record” (39–41). However, even the most elaborate information-sharing tool might not be able to alleviate the unease und uncertainty that is associated with the reflection of normative social processes challenged by variations in sex development (36). Psychological support is essential.

Since March 2021 German legislation bans cosmetic genital surgery on children with a DSD diagnosis (42). Genital interventions in children with DSD who are too young to consent can be authorized in individual cases by a family court and require the documentation of comprehensive multidisciplinary education and care during decision-making (14). This change occurred during the Empower-DSD study. It has led to the postponement of elective surgery to modify genital appearance to the age when the children are mature enough to make decisions. However, rushed decisions for early interventions in childhood are not necessary for the majority of children with DSD. The focus instead lies on ongoing information transfer and comprehensive education of the child and parents. The information management program can assist this process.

### 4.5 Organization of peer counseling, case conferences and collaboration with primary care

In the quantitative assessment of the checklists, it was noticeable that peer counseling, case conferences between centers, and counseling on social and legal aspects were documented less frequently than other elements of care. In the qualitative interviews, parents reported an interest for peer counseling but were also overwhelmed by the appointments and information in the DSD clinic during the initial phase after diagnosis. Therefore, peer support might be sought later on. Although, it is a known phenomenon that many families do not seek peer support (21). Written narratives from peers with DSD might facilitate contacting peer support groups and could be included in the information material of “my record”. Reports from parents with children with DSD are available in English and could be translated to German (43–45). Written reports from adults with DSD are available online (46, 47). In-house peer counseling was successfully established in one DSD center and could be integrated in the information management program to promote peer contact (48). Following the Empower-DSD study, case conferences between centers in Germany were continued in the national project DSDCare, which ended in 2023. Now virtual consultations as part of a Clinical Patient Management System are offered regularly via the Endo-ERN network (49).

Another care element in the information management program was the collaboration with primary care. Nevertheless, feedback to the referring provider was rarely documented in the checklist. A form to document the handover might optimize care.

### 4.6 Strengths and limitations

One strength of the study was the involvement of different partners. Self-help groups and professionals cooperated in the development of the materials, the design of the evaluation and implementation of the information management program. This ensured that the interests and needs of the families and children were considered at every step of the program's development and implementation. In the evaluation, the large number of included families and the use of the mixed methods approach allowed there to be an inclusion of different perspectives on DSD care. This is especially important as preferences in clinical care can vary depending on individual preferences. In a recent publication there has been attempts to define characteristics of good DSD care by interviewing stakeholders (children, families, and health care providers) (50). There was strong agreement that patient satisfaction, mental and physical health, and satisfaction with social gender were the most important outcomes. The importance of functionality, external genital appearance, and sexual function were rated differently. The approaches used to achieve the goals of good care, such as postponing unnecessary medical interventions until the child is able to make an informed decision or the parents making decisions, were rated as being of varying importance. These findings show that the values and goals of each family are unique and should be explored and considered by care providers. The majority of the included children in this study were infants. Therefore, little data on youths can be reported. This is a limitation of the study reported in this paper. Future studies should examine whether older children are less often diagnosed in specialized DSD centers. There should also be an evaluation of whether the needs of older children in the first weeks after a DSD diagnosis differ from of the needs of parents with younger children. A further limitation of the study is that checklist completion relied on MDT report. Within the Empower-DSD study there was no external supervision to validate the completion of the checklists.

It was not specified in advance which member of the MDT should complete the checklist. It is not clear if the missing elements in the checklist were omitted or wrongly recorded. This is a methodological limitation of the present study.

## 5 Conclusion

The evaluation shows that the structured care and materials within the Empower-DSD information management program were mostly perceived to be valuable. Positive feedback was given for the developed care pathway, materials, and the resulting trusting relationship. Multiple resources (time, accessibility) are required by all involved stakeholders to ensure a good quality of information exchange and optimal care of the children and their parents. Early access to specialized care is important for families and has to be further improved. The structured support within the first weeks after a DSD diagnosis can start a process of information exchange and provide the basis for life-long care. It can support the process of children developing into satisfied young adults who are well informed about their diagnosis. Information on the nature of the decision-making process and peer narratives could be added to the information material. Further studies should evaluate the long-term impact of the information management program on coping and wellbeing of children with a DSD diagnosis.

## Data Availability

The raw data supporting the conclusions of this article will be made available by the authors, without undue reservation.
