# Supplementary material for: Structured care after a DSD diagnosis in childhood: a mixed methods evaluation of the Empower-DSD program
Source: Front Pediatr. 2025 Mar 24;13:1488411. doi: 10.3389/fped.2025.1488411 (PMC11973257; doi:10.3389/fped.2025.1488411)
Supplement: Supplementary file 1 [file Datasheet1.pdf]

Sample-Checklist

Study-ID
Sample

Age: * 04/2021

Diagnosis: 46 XY-DSD

First Visit

Date: 13.04.2021

Check with x if completed, otherwise leave blank

- o Explain goal of information management.
- o Introduce team members.
- o Concept of psychosocial care
- o Concept of peer counselling
- o Hand out „my record".
- o Outline diagnostic process.

Diagnostic process

x…completed o… not completed xx…previously done n … not applicable

	Date	Date	Date	Date	Date	Date	Date
	13.04 2021	26.04 2021	19.10 2021	01.12 2021			
Medical history	X	X	X	X			
Somatic assessment	X	O	X	O			
Biochemical and genetic assessment	X	O	X	O			
Sonography / imaging	X	O	X	O			
Referral gynacology	O	O	n	O			
Referral urology	O	O	X	O			
Referral pediatric surgery	O	O	O	O			

Diagnosis

c…confirmed u…unclear s…suspected

Diagnosis	c	c	c	c			

Discuss results, medical information

x…topic discussed o…not discussed n…not applicable

Basics: Sex development, gonads, steroid hormones	X	X	X	X			
Urgency or non-urgency of treatment	n	n	n	n			
Development of gender identity	X	X	X	O			
Expected somatic development	O	X	X	O			
Options for hormone treatment in puberty	O	X	X	O			
Individual decision making	O	O	X	O			
Fertility	O	X	X	O			
Malignant potential	O	X	X	O			
Sexuality	O	X	X	O			
Risks and benefits of surgery	X	X	X	O			
Management plan	O	O	X	O			

Psychosocial care parents

Address questions, fears, and concerns	X	X	X	O			
Adopt a positive perspective on the child´s future	X	X	X	O			
Each family has a different way to approach DSD	X	O	X	O			
Recommend to document decision-making process	O	X	X	O			
View of biological sex and gender as continua	O	O	X	O			
Diversity of physical appearance	X	O	X	O			
Sex assignement	X	O	X	O			
Communication within the social network	X	X	X	O			
Communication with the healthcare system	X	X	X	O			
Explain DSD to the child in an age-appropriate way	n	n	n	O			

Psychosocial care child

Address questions, fears, and concerns							
Adopt a positive perspective on the future							
Education about the child´s body and condition							
Diversity of physical appearance							
Communication with the social network							
Explain procedures in the specialized center							
Explain care and guidance over time							

infant

Gefördert durch: Gemeinsamer Bundesausschuss Innovationsausschuss

	x…topic discussed o…not discussed n…not applicable					
Social and legal issues	O	X	X	O			
Peer counselling	O	O	X	O			

	In the center<br>Date	Cross-center<br>Date	
Case conference	19.01.2021		
Professions attending	*x…present o…not present n…not applicable*		
Pediatric endocrinology	X		
Psychology	X		
Social work	O		
Pediatric Gynacology	O		
Pediatric urology	X		
Pediatric surgery	O		
Pediatric radiology	O		
Genetics	O		
Other (e.g. Ethics)	O		
**Center**	*x…present o…not present n…not applicable*		
Berlin Charité	O		
Bochum UKRUB	X		
Lübeck UKSH	O		
Münster UKM	O		
UK Ulm	O		
Topics	*x…topic discussed o…not discussed n…not applicable*		
Results of assessments	X		
Urgency of treatment	n		
Evidence for recommendations	X		
Management plan	X		

Feedback to referring primary care provider Datum: /

Gefördert durch:
Gemeinsamer Bundesausschuss
Innovationsausschuss
