# Supplementary material for: Structured care after a DSD diagnosis in childhood: a mixed methods evaluation of the Empower-DSD program
Source: Front Pediatr. 2025 Mar 24;13:1488411. doi: 10.3389/fped.2025.1488411 (PMC11973257; doi:10.3389/fped.2025.1488411)
Supplement: Supplementary file 2 [file Datasheet2.docx]

**Interview guide for parents EMPOWER-DSD Information management program**

The questions/narrative impulses marked with a dot are thematic core questions, the questions marked with a dash can (but do not have to) be used for follow-up questions.

**Introduction**

I am very pleased that you have agreed to be interviewed as part of this study. Do you have any questions before we begin?

Then I will now start the recorder. *(Start recording device)*

Your child has been diagnosed with a variant of sex development. At the beginning of the interview, it is important for me to know how you describe the variation and how we should refer to it in this interview.

**Information management program and support**

- You and your family have regular appointments at the XY Center and are therefore taking part in what is known as an information management program. what are your experiences and what do you think of the program
- How do you feel about the care you receive? Can you give me examples of this?
- How are your concerns and needs addressed?
- What would you change or wish for in the care you receive at Center XY (the information management program)?
  - Was there anything you did not like?

Effects

- What effects have you experienced on your handling of the diagnosis since you have been receiving care at Center XY?
- What influence did the care you received there have on your treatment decision?
  - How informed do you feel since then?

Expectations and wishes

- When you think back: what expectations and wishes did you have when you came to the XY Center for further care?

**Dealing with the diagnosis/suspected diagnosis**

- - I would like to talk about what it was like for you and your family when you received the diagnosis/suspected diagnosis: If you think back, what was it like?
  - How did you and your family deal with it?
  - Who did you talk to about it?
  - Who or what helped and supported you?
  - What was difficult for you?
  - Has anything changed for you and your family in dealing with the diagnosis since then? If so, what? Please give me examples.
  - What would you generally consider to be good support for families with a new diagnosis?
  - What would you wish for?

Puberty

*(only if child with DSD is in puberty)*

- - Your child was diagnosed at puberty. The transition from child to adult care is a time that is accompanied by many changes. How are you experiencing this right now?
  - What are you particularly concerned about?

**Conclusion**

- - Is there anything else you would like to add that is important to you but has not yet been mentioned?
  - Do you have any questions for me?

Thank you very much for the interview.
